# Pulp Digestion

**Published:** 1901-03-15

**Authors:** A. W. Harlan

**Affiliations:** Chicago, Ills.


### PULP DIGESTION.

BY A. W. HARLAN, A.M., M.D., D.D.S., CHICAGO, ILLS.

Read before the International Dental Congress at Paris, August 8, 1900.

In presenting to your notice the subject of pulp digestion it is my aim to provide a method for removing from the roots of teeth dead pulp tissue which, through lack of skill or lack of operative technique for its thorough removal, is ordinarily allowed to remain. Many methods of obviating the necessity for pulp removal have been presented to learned societies, all based on the hypothesis of pulp mummification, and that subject is covered in other communications presented to the congress.

In looking over the subject of mummification I do not find in the department of surgery any analogy for such procedure as a permanent operation.

I conclude that the destruction of pulps by any method means their removal from the roots before it is a safe surgical procedure to fill them. If this proposition be true, I desire to call your attention to a process for the liquefaction of pulp tissue in small, tortuous roots and those not easily accessible.

In times past the digestion of animal tissue has been accelerated by the use of pepsin or some of its preparations, and of starchy matters by taka-diastase. In the year 1881 Van Antwerp, of Kentucky, discovered that the fresh leaves of pawpaw, or *Carica papaya*, would digest fresh meat, beefsteak, and other animal flesh.

The pulp, as is well known, consists of blood vessels and other tissues, and is dissimilar to any other formation in the body. It is capable of coagulation, and will undergo putrefaction after death, the same as others of the soft tissues. This being granted, we must at once concede that there are teeth having very attenuated and twisted roots where it is almost a physical impossibility to remove all the dead pulp tissue before the putrefactive process begins.

To relieve us of this operative procedure, I have long been interested in the digestion of such tissue and will present now the experiments to show the possibility of liquefying such tissues in teeth.

Papain (papayatin), which is obtained from the *Carica papaya*, is now procurable from such manufacturers as Merck, Schuchardt, and others. It is a whitish, somewhat hygroscopic powder, having the taste of pepsin. It is soluble in water and glycerol. It is active in an acid, neutral, or faintly alkaline solution. It has been used notably in general medicine, diphtheria, in fissures and ulcers, and generally in the domain of surgery as a digester. In stomachic disturbances the best results have been obtained. It is absolutely non-irritating; it is a non-coagulator, and without action on living tissue. The fresh leaves of the pawpaw have long been noted in our country for their softening effect on tough meat, the joints of fowls, etc. My first experiences with pawpaw began more than three years ago. The first requisite in its successful use is to have the pulp dead. It does not act well when made into a fresh paste with water unless it is slightly acidulated with hydrochloric acid, 1 to 300. Its action is slower when a dead pulp has been treated with acids, creasote, carbolic acid, or zinc chlorid. When oils have been rubbed on the surface or arsenical paste has been in contact with a pulp its solution or digestion is not retarded.

I find that pulp tissue is digested more rapidly if the surface has been lightly painted with 1 to 200 sodium fluosilicate, or a solution of borate of soda, 1 to 200; carbonate of soda or magnesia may be used, but not in excess.

If one gram of papain is made into a thick paste with glycerol and a drop of hydrochloric acid solution, 1 to 300, is added, it always acts well. My experiments out of the mouth were conducted as follows:

Freshly extracted teeth were taken and the roots enveloped in cotton soaked in distilled peppermint water; the pulp chamber was opened and the first pulp had the paste applied to it without first touching it with any drug. The remainder were touched with alcohol, zinc chlorid, arsenical paste, oil of cassia, creasote, carbolic acid, and sulfuric acid. As much of the paste was packed into the pulp chamber as it would hold, and the cavity was sealed and the roots kept moist in a chamber where the temperature was even at 98½ degrees Fahrenheit. These cases were examined at the expiration of twenty-four hours without sensible effect. They were again examined in forty-eight, seventy-two, and ninety-six hours. Complete solution would not take place under ninety-six hours, and if there was considerable pulp tissue in the root it was usually five to six days before solution would take place.

When the roots of the teeth were enveloped in liquid vaselin or in distilled water to which half of one percent of peppermint or cassia had been added, the results were almost identical. During the past winter and spring months I used about one hundred and fifty freshly extracted teeth, but found it impossible to digest fragments of the pulp in small or tortuous roots under five to six days. In the mouth, clinically, the papain will act more rapidly, four days being usually sufficient to digest small fragments of pulp tissue. If a large mass of pulp tissue remains in a tooth the papian paste must be reapplied at the end of the second day, and occasionally for a third time. I have found from repeated experiments that it is best to remove all tissue in sight, and then pack the roots and pulp chamber full of the paste and seal with oxysulfate or oxyphosphate of zinc, and let it remain without interference five to eight days. In this way the patient suffers no uneasiness, and there is no risk of pericemental irritation. Putrefaction does not take place in the presence of a solution made in glycerol.

It is needless to remark that this operation must be done under antiseptic precautions from beginning to finish.—*Dental Cosmos.*

